# Examining seasonal effect of urban heat island in a coastal city

**DOI:** 10.1371/journal.pone.0217850

**Published:** 2019-06-14

**Authors:** Xiangli Wu, Lin Zhang, Shuying Zang

**Affiliations:** 1 Key Laboratory of Remote Sensing Monitoring of Geographic Environment, College of Heilongjiang Province, Harbin, Heilongjiang, China; 2 School of Geographical Sciences, Harbin Normal University, Harbin, Heilongjiang, China; University of Wisconsin Milwaukee, UNITED STATES

## Abstract

Urban heat islands (UHIs) have a significant and negative impact on the urban ecological environment and on human health, and it is imperative to examine factors that lead to UHIs. Although numerous studies have been conducted in this field, little research has considered seasonal variations in UHIs in coastal cities. Moreover, parametric statistical analyses, such as regression and correlation analyses, have been typically applied to examine the influential factors. Such analyses are flawed because they cannot uncover the complicated relationships between UHIs and their factors. Taking Dalian, a coastal city in China, as an example, this paper reveals the dynamic mechanism of the UHI effect for different seasons using the cubist regression tree algorithm. Analyses suggest that the UHI effect only exists in spring and summer, and no obvious UHIs can be found in autumn and winter. The adjacency to the sea leads to moderate UHI effects in spring and summer and no UHI or urban cooling island (UCI) effects in autumn and winter. The distance to the coastline, however, does not play a role in the UHI effect. Furthermore, as one of the most important factors, the vegetation coverage plays a significant role in the UHI effect in spring and summer and significantly mediates the UHI in autumn and winter. Comparatively, the elevation (e.g., digital elevation models (DEMs)) is consistently negatively associated with the land surface temperature in all seasons, although a stronger relationship was found in spring and summer. In addition, the surface slope is also a significant factor in spring and winter, and the population density impacts the UHI distribution in summer as well.

## Introduction

The urban heat island (UHI) effect, which refers to higher temperatures in urban areas than in surrounding rural areas, has significant and negative impacts on the urban ecological environment and human health [1,2]. Since it was first recorded by British meteorologist Manley in 1958 [1], the UHI effect has become an important topic, and numerous studies have been conducted to examine factors that lead to UHI effects. Urban biophysical compositions, particularly vegetation, urban impervious surfaces, and soil, have suggested to be highly associated with UHIs [3–6]. In addition, building infrastructure and adjacent heat sources have been suggested as factors that affect UHIs [7]. Similarly, aggregations and orientations of urban buildings have been identified to have different effects on the distribution of UHIs [8]. Urban traffic route planning and building roof materials have also been identified as factors affecting UHIs [9,10]. Although numerous studies have been conducted to study the major factors related to the UHI effect, existing research on UHIs emphasizes inland cities, and little research has been applied to coastal cities. For a coastal city, the effects of sea are likely to play a role in mitigating the UHI effect, as the temperature variations in water are relatively small compared to built-up materials. Moreover, seasonal variations in the UHI effect are ignored in the existing literature, as most studies have attempted to examine the effects of urbanization and population growth on the UHI effect. That is, the same season was always used for a comparative analysis. Furthermore, existing studies typically applied parametric statistical analysis approaches, such as correlation and regression analyses, to examine the influential factors. Such techniques, however, are flawed because they cannot reveal the complicated relationships between UHIs and their major influential factors. To address the aforementioned problems, this research proposes to answer three questions, including 1) whether the UHI effect in a coastal city is significantly different from that in an inland city, that is, whether the adjacency to the sea affects temperature variations; 2) for different seasons, whether the influential factors are significantly different and whether there is a significant cooling effect in winter; 3) whether a nonparametric approach, e.g., classification and regression tree algorithms (e.g., the cubist algorithm), is more appropriate for UHI studies.

## Materials and methods

### Study area

**Fig 1:**
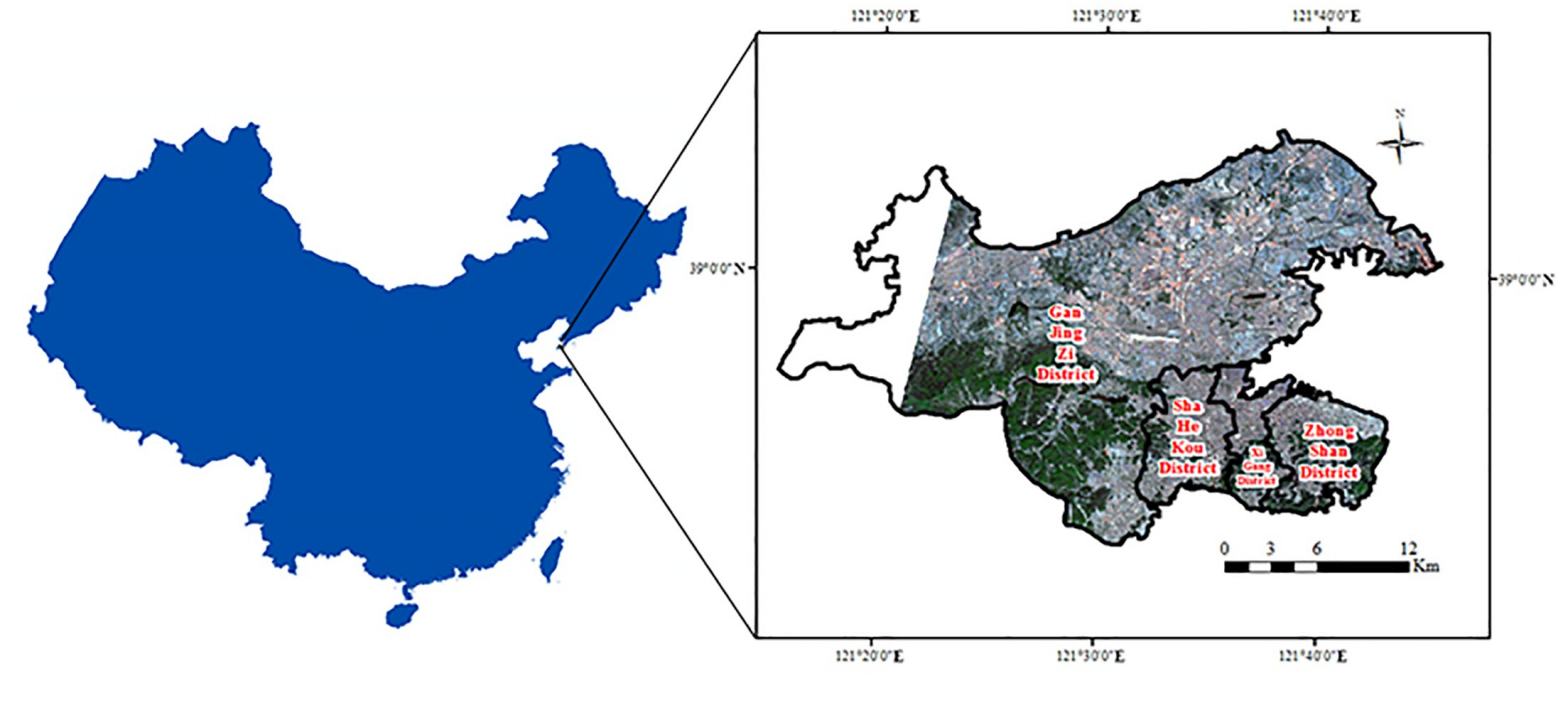
Dalian City, Liaoning Province, China.

Dalian, a coastal city at the southern tip of Liaodong Peninsula in Liaoning Province, China, was selected as the study area. Dalian lies between 38°50’27”N and 39°05’10”N latitude and between 121°20’57"E and 121°45’22”E longitude (Fig 1). It is located south of the Bohai Sea and north of the Yellow Sea. The administrative area of Dalian includes the main city, Lüshunkou District, Jinzhou District, Zhuanghe, Pulandian District, and Wafangdian, covering almost 13,237 km^2^. In this paper, we chose the main city (including Zhongshan District, Xigang District, Shahekou District, and Ganjingzi District) as the study area. The main city of Dalian is covered by low hills with higher elevations in the north and a few plains with lower elevations in the south. Dalian is in the warm temperate climate zone, with temperate monsoon climate and oceanic characteristics. It has a mild climate with four distinct seasons. In summary, Dalian has complex terrains, an obvious maritime climate, a high population density, and complex building layouts.

### Data source and preprocessing

According to the division of the Dalian Meteorological Bureau, March-May, June-August, September-November, and December-February are defined as spring, summer, autumn, and winter, respectively. To examine the UHI effect, we adopted Landsat 8 imagery as the major data source because of its large coverage and relatively high spatial resolution [11]. In this paper, we selected four cloudless Landsat 8 Thermal InfraRed Sensor (TIRS) images on May 8, August 28, November 16 and February 2 during 2016 as the data source for the four seasons (Table 1). In addition to remote sensing imagery, air temperature data were also obtained from the meteorological station in Dalian City for validation purposes. We also downloaded the digital elevation data with a resolution of 30 m (strip number 121, line numbers 38 and 39) from the United States Geological Survey (USGS) website (https://www.usgs.gov/). Furthermore, we downloaded night Defense Meteorological Satellite Program/ Operational Linescan System (DMSP/OLS) light data via the United States National Geophysical Data Center website (http://www.ngdc.noaa.gov). Night light data include three types of annual average data: average visible, stable lights (SL) and cloud-free coverages (cloudless observation). Since most data were limited to 2013, this paper employed the 2013 global nighttime light values as the proxy for the 2016 stable light (SL) data. For the SL data, the background value is 0, and the light intensity range is 1~63.

**Table 1:** Surface temperature statistics in 2016.

Season	Date	Max	Min	Mean	SD
Spring	May 8, 2016	43.44	10.58	28.1	3.29
Summer	August 28, 2016	48.18	23.1	33.76	3.45
Autumn	November 16, 2016	18.49	1.97	11.08	1.12
Winter	February 2, 2016	10.69	-7.66	2.3	1.85

### Methods

#### Surface temperature inversion

For generating surface temperature information from thermal infrared bands, common methods include Jiménez-Munoz’s single-channel algorithm [12], Qin’s single-window algorithm [13], Rozenstein’s split-window algorithm [14] and the atmospheric correction method (radiation transmission equation method). Previous research has shown that Qin’s single-window algorithm is suitable for Landsat TIRS bands [15]. While Landsat 8 has both the 10th and 11th thermal infrared bands, the USGS recommends employing the 10th band for single-band thermal infrared data because of its optimal stability [16]. In this paper, a modified single-window algorithm is applied to the geothermal inversion of the 10th band of the Landsat 8 TIRS data.

$$T_{s}\;=\;\left[a(1-C-D)+(b(1-C-D)+C+D)T_{10}-DT_{a}\right]/C$$ (1)

where *T*_*s*_ is the land surface temperature for a pixel, *a* and *b* are parameters (*a =* -62.735657, *b =* 0.434036), *C* and *D* are parameters relevant to atmospheric transmittance (*τ*), *T*_*10*_ is the brightness temperature of Band 10 (10.6 μm~11.19 μm), and *T*_*a*_ is the average temperature of the atmosphere.

With Eq (1), the parameters *C* and *D* are calculated as follows:

C=δτ (2)

D=(1-τ)[1+(1-δ)]τ (3)

where *τ* is the atmospheric transmittance (*τ = P*_*1*_*w3+P*_*2*_*w2+P*_*3*_*w+P*_*4*_), with *P*_*1*_ *=* 3.746415*×*10–3, *P*_*2*_ = –3.988729*×*10–2, *P*_*3*_ = –5.00628*×*10–3, and *P*_*4*_ = 0.947512; *w* is atmospheric water vapor content; and *δ* is the surface emissivity.

Further, the average atmospheric temperature *T*_*a*_ was estimated using the equations illustrated in Table 2. In this paper, we chose the midlatitude summer model to estimate *T*_*a*_. For calculating *T*_*a*_, we obtained the atmospheric profile information of atmospheric humidity, pressure and atmospheric temperature from the National Aeronautics and Space Administration (NASA) website (http://atmcorr.gsfc.nasa.gov). The average atmospheric temperature can be estimated from the near-surface temperature *T*_*0*_.

**Table 2:** Equations for estimating the average operation temperature of the atmosphere.

Atmospheric model	Average atmospheric temperature estimation equation
Tropical Atmosphere	*T_a_* = 17.9769+0.91715*T0*
Midlatitude Summer	*T_a_* = 16.0110 + 0.92621*T0*
Midlatitude Winter	*T_a_* = 19.2704 + 0.91118 *T0*
1976 U.S. Standard Atmosphere	*T_a_* = 25.9396 + 0.88045*T0*

Furthermore, we employed the method developed in [12, 17] to estimate the emissivity *δ*.

$$\delta \;=\;T_{A}P_{v}+T_{b}(1-P_{v})$$ (4)

where *δ* is the surface emissivity, *T*_*A*_ denotes the emissivity of natural surfaces, *T*_*b*_ denotes the emissivity of urban surfaces, and *P*_*v*_ represents the vegetation coverage. The remote sensing image was classified into three land cover types: water bodies, urban areas and natural surfaces. The emissivity of water was set to 0.99, and the emissivity of natural surface (*T*_*A*_) and urban surface (*T*_*b*_) were calculated via the following formulas:

$$T_{A}\;=\;0.9625+0.0614P_{v}-0.0461P_{v}^{2}$$ (5)

$$T_{b}\;=\;0.9589+0.086P_{v}-0.0671P_{v}^{2}$$ (6)

respectively.

For this paper, the vegetation coverage (*P*_*v*_) was calculated following the approach developed in [13, 18, 19] (see Eq (3)).

$$P_{v}\;=\;{\left[\left(NDVI-{NDVI}_{Soil}\right)/\left({NDVI}_{Veg}-{NDVI}_{Soil}\right)\right]}^{2}$$ (7)

where *Pv* is vegetation coverage, *NDVI* is the normalized difference vegetation index, *NDVI*_*soil*_ is the NDVI threshold for the complete bare-soil or vegetation-free area, with a value of 0.05. *NDVI*_*Veg*_ is the fully vegetated NDVI threshold, with a value of 0.7. For this paper, the *NDVI* was calculated using the digital numbers of OLI bands 3 and 4.

Finally, *T*_*10*_ was calculated using the following equation:

$$T_{10}\;=\;\left(K_{2}\right)/ln\;\left(1+K_{l}/L_{\lambda }\right)$$ (8)

where *T*_*10*_ is the brightness temperature, and *T*_*s*_ is the land surface temperature. *K*_*1*_ and *K*_*2*_ are the moduli for the Landsat 8 TIRS 10th band, where *K*_*1*_ = 774.89 W/(m^2^·μm·sr), and *K*_*2*_ = 1321.08 K.

#### Classification of the surface temperature

To further analyze the UHI effect, we divided the temperature into a few zones using the average (*a*) and standard deviation (*S*_*d*_) of the surface temperature as the cutoff criteria. We used the equally spaced grading method. We divided the surface temperature into six levels (Table 3).

**Table 3:** Determination of the ranges of different surface temperature intervals.

Temperature zone	Range
Low temperature zone	*T*_*s*_ < *a*—*S*_*d*_
Secondary low temperature zone	*a*-*S*_*d*_ ≤ *T*_*s*_ < *a*-0.5*S*_*d*_
Medium temperature zone	*a—*0.5*S*_*d*_ ≤ *T*_*s*_ ≤ *a*
Secondary high temperature zone	*a* ≤ Ts ≤ *a* + 0.5*S*_*d*_
High temperature zone	*a*+0.5*S*_*d*_ ≤ *T*_*s*_ ≤ *a*+*S*_*d*_
Extremely high temperature zone	*T*_*s*_ > *a*+*S*_*d*_

*T*_*s*_: surface temperature; *a*: average surface temperature; *S*_*d*_: standard deviation of the surface temperature

#### Distance to coastlines

**Fig 2:**
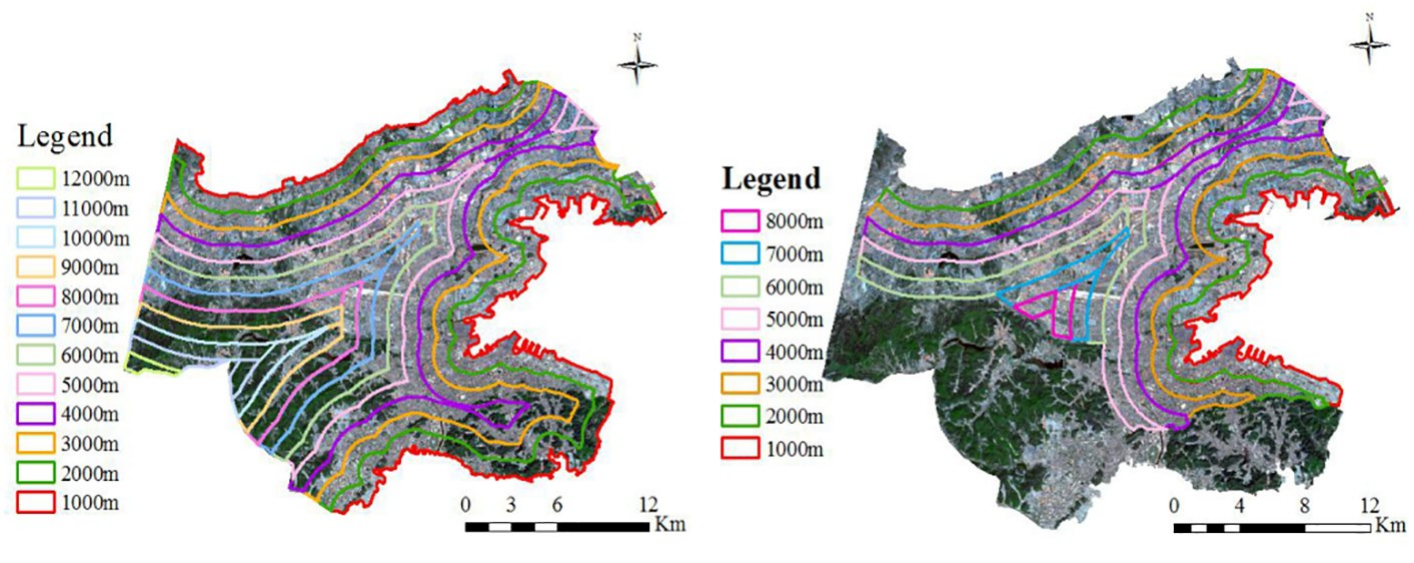
Buffer map of the study area (left) and the buffer map without the vegetated and mountainous areas (right).

To examine the impact of sea on Dalian’s UHI effect, we established eight 1000 m buffer zones from the coasts to the interior of the study area, and the distance to the coastline was extracted using the Euclidean distance method. Because both the vegetated and mountainous areas may impact the UHI, we removed the vegetated and mountainous areas to analyze the coastal effect (Fig 2).

### Cubist model

**Fig 3:**
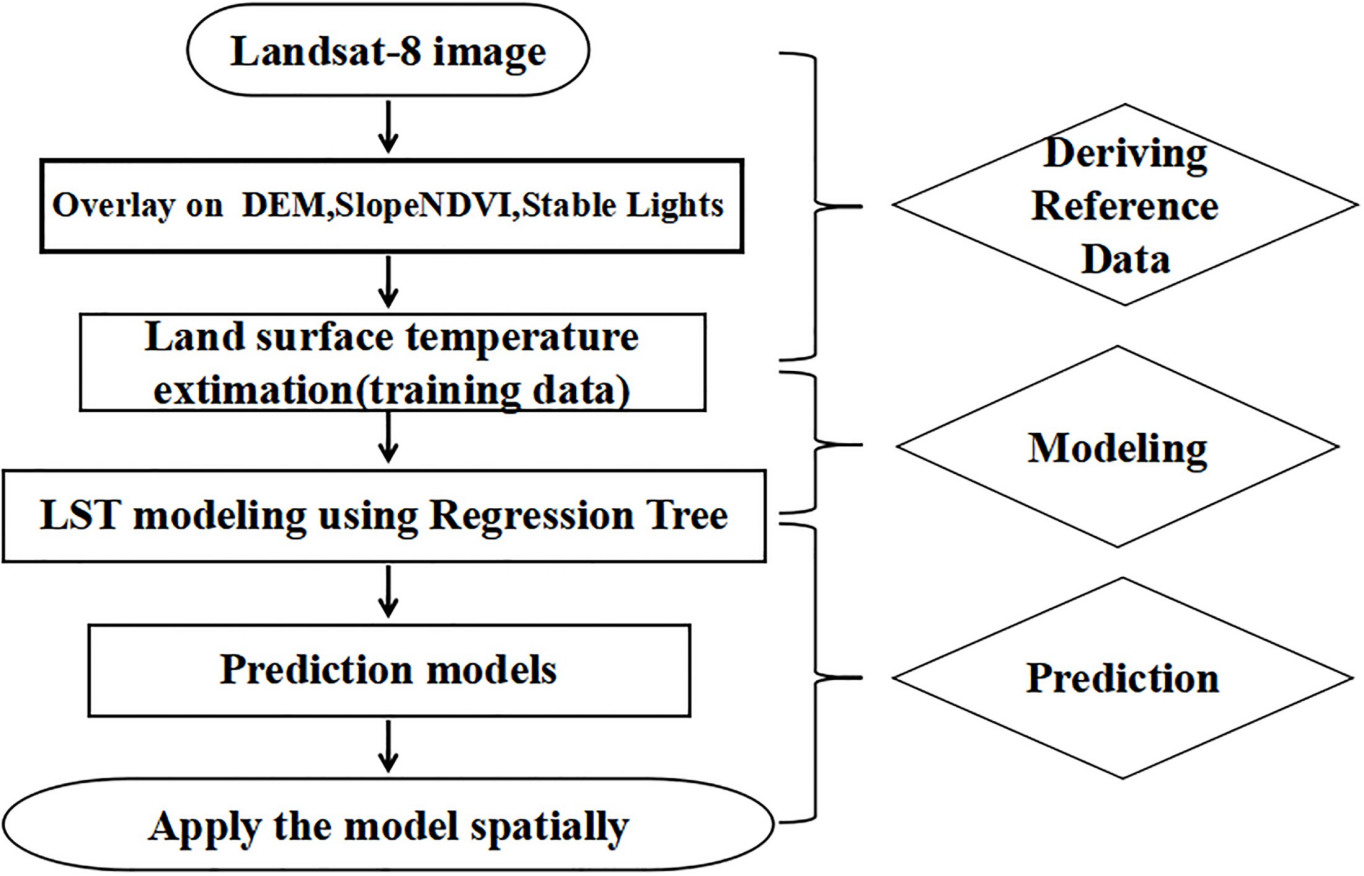
Flowchart of the proposed method.

To analyze the relationship between the UHI effects and environmental and socioeconomic factors, we adopted a regression tree analysis model. The regression tree algorithm produces rule-based models for the prediction of continuous variables based on training data [20]. Each rule set defines the conditions under which a multivariate linear regression model is established. Regression tree models can account for a nonlinear relationship between the independent and dependent variables and allow both continuous and discrete variables as input variables. The cubist algorithm, a regression tree algorithm (detailed information on the cubist software is available at http://rulequest.com/cubist-info), was employed to examine the relationship between UHIs and their factors. According to specific rules, the algorithm grows a categorical and binary tree by repeatedly splitting the data into subsets, depending on how the dependent variable and the independent variables interact [21]. It uncovers the predictive structure of the problem under consideration to categorize the data into more homogeneous subsets [20]. For each subset, a multivariate linear regression model is constructed, and the splitting rules are specified such that the combined regression model residual errors of each subset are substantially lower than that of the single best model before partitioning [22]. It has been reported that the accuracy and predictability of regression tree models are better than those of simple linear regression models [23]. Another feature of the cubist algorithm is its ability to estimate the predictive accuracy via an n-fold cross-validation. Using this option, the training data set can be divided into n blocks of roughly equal size. For each block in turn, a model was built from the data in the remaining blocks and tested using the holdout blocks. The final accuracy of the model was estimated by averaging the model results from all the n-fold tests [24]. Here, we employed the cubist algorithm to analyze the relationship between the urban heat island effect and its physical and environmental factors. The major steps of this method are as follows (Fig 3).

## Results

### Seasonal temperature distribution characteristics

**Fig 4:**
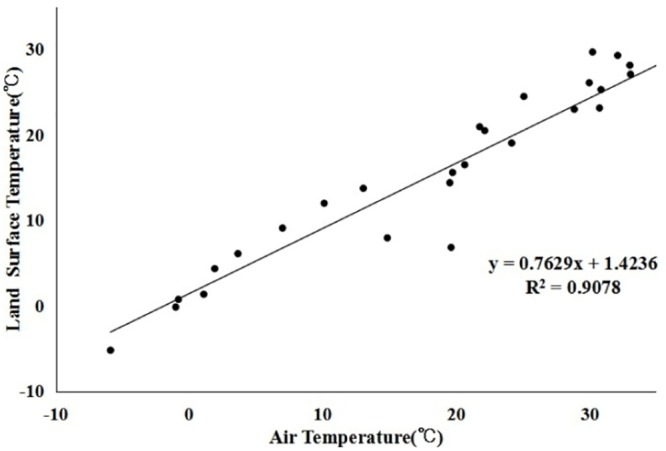
Scatter plot of the measured air temperature and the remotely sensed land surface temperature.

We verified the derived surface temperature via the measured air temperature (provided by the Dalian Meteorological Monitoring Station). Although the measured data cannot completely represent the surface temperature of all the pixels, the data can be indirectly employed as an accuracy test for the inversion result. Because there was only one measured point (38.54°N, 121.38°E) in the study area, we chose the data from the last four years with cloudless dates (28 days in total) for verification. Because the images were acquired at 10:30 a.m. local time, we employed the measured temperature data obtained from the Dalian Meteorological Monitoring Station at 10:30 a.m.as the ground truth values. The accuracy verification (Fig 4) showed that the measured air temperature and the inverse of the land surface temperature fit very well, with an R^2^ of 0.9078 (the very large value of R^2^ may be because the 28 points are from the same site: 38.54°N, 121.38°E). Therefore, we infer that the single-window algorithm is suitable for this study.

**Fig 5:**
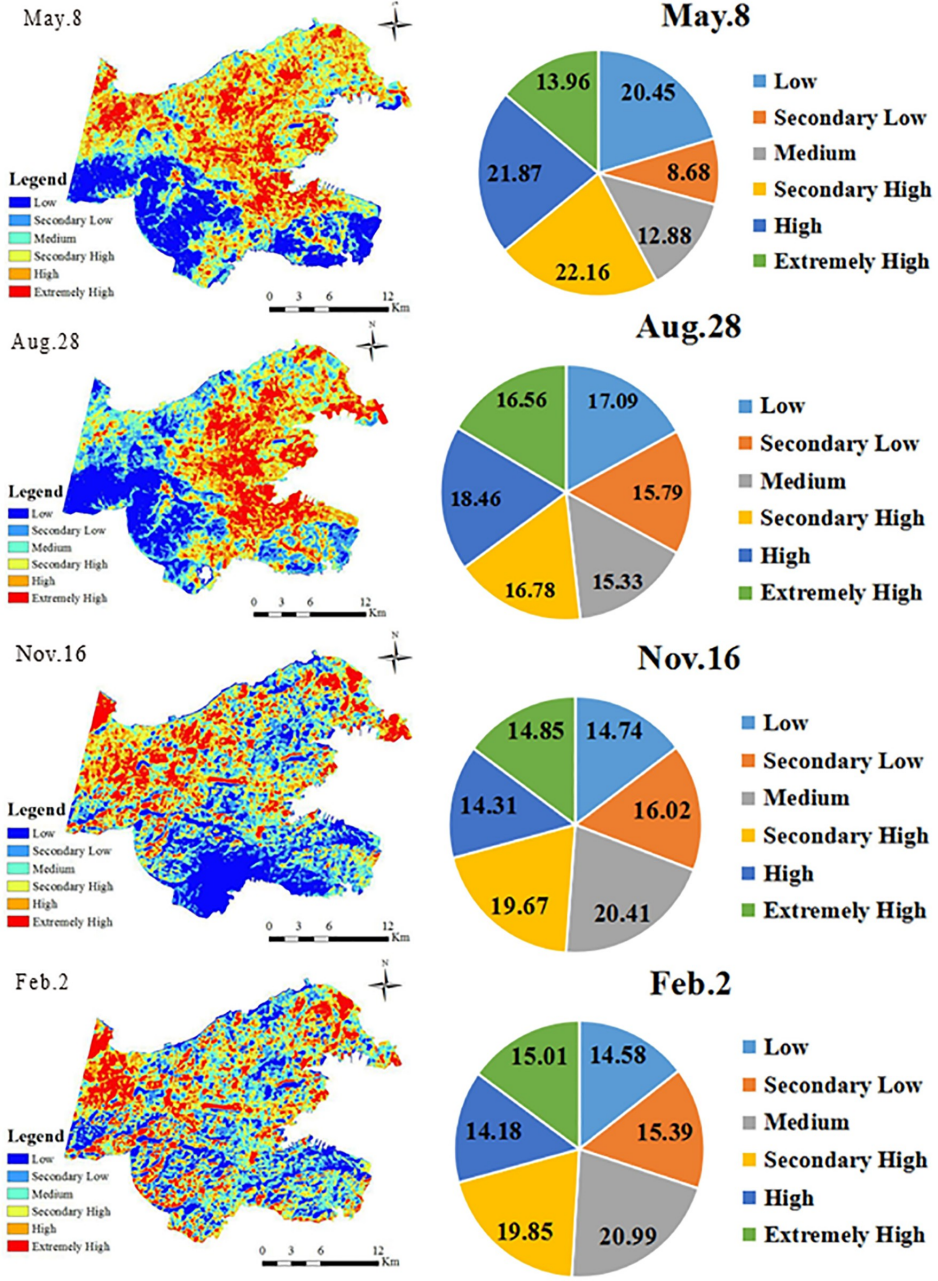
Spring, summer, autumn and winter temperature distributions and percentages for each temperature zone.

The estimated land surface temperatures for the four seasons are shown in Fig 5 and Table 4. From Fig 5, the UHI effect in Dalian City in the spring and summer is significant, because the high temperature zones are generally consistent with the urban built-up areas near the harbor area of Zhongshan District and the densely built-up areas in the downtown areas of Shahekou District and Ganjingzi District. The intensity of the UHI, however, is relatively low. In summer, the high temperature zone and the extremely high temperature zone occupy approximately 18.46% and 16.56% of the total study area, respectively. Furthermore, in spring, areas with high and extremely high temperatures are approximately 21.87% and 13.96% of the total study area, respectively. The relatively low intensity of the UHI may be due to the ocean effect, in which the sea-land breeze prevails, and the lower temperature in mornings and evenings.

**Table 4:** Surface temperature statistics (°C).

Date	Max	Min	Mean	SD
**May 8, 2016**	43.44	10.58	28.1	3.29
**August 28, 2016**	48.18	23.1	33.75	3.45
**November 16, 2016**	18.49	1.97	11.08	1.12
**February 2, 2016**	10.69	-7.66	2.3	1.85

For autumn and winter, however, the UHI and the urban cooling island (UCI) effects do not exist, as the surface temperatures are similar across Dalian, with relatively higher temperatures in the northwestern region: a suburban area. This result may be associated with the ocean effect. The heat capacity of water is relatively large, and the process of heat absorption and dissipation on the surface of the sea is slow. Moreover, Dalian belongs to a temperate monsoon climate, thereby leading to gentle temperature changes due to the ocean effect. Compared with inland cities at the same latitude, Dalian has a mild maritime climate, and each temperature zone is evenly distributed. This finding is consistent with the results obtained in [25, 26], which reported that the spring and summer heat island phenomena are obvious in Chicago, IL, United States. Another possible explanation could be the suburban development policy of Dalian, which led to a large number of people moving to this suburban area, together with factories, especially heating companies. As a result, a large amount of manufactured heat may be produced in the suburban areas in the northwestern region because of the relocation of factories.

### Analyses of factors affecting the UHI

We further examined the influential factors, including the elevation and slope, the NDVI, the distance to the coastline, and the stable light, on the UHI effect. The results of these analyses are detailed as follows.

#### Elevation (DEM) and slope

**Fig 6:**
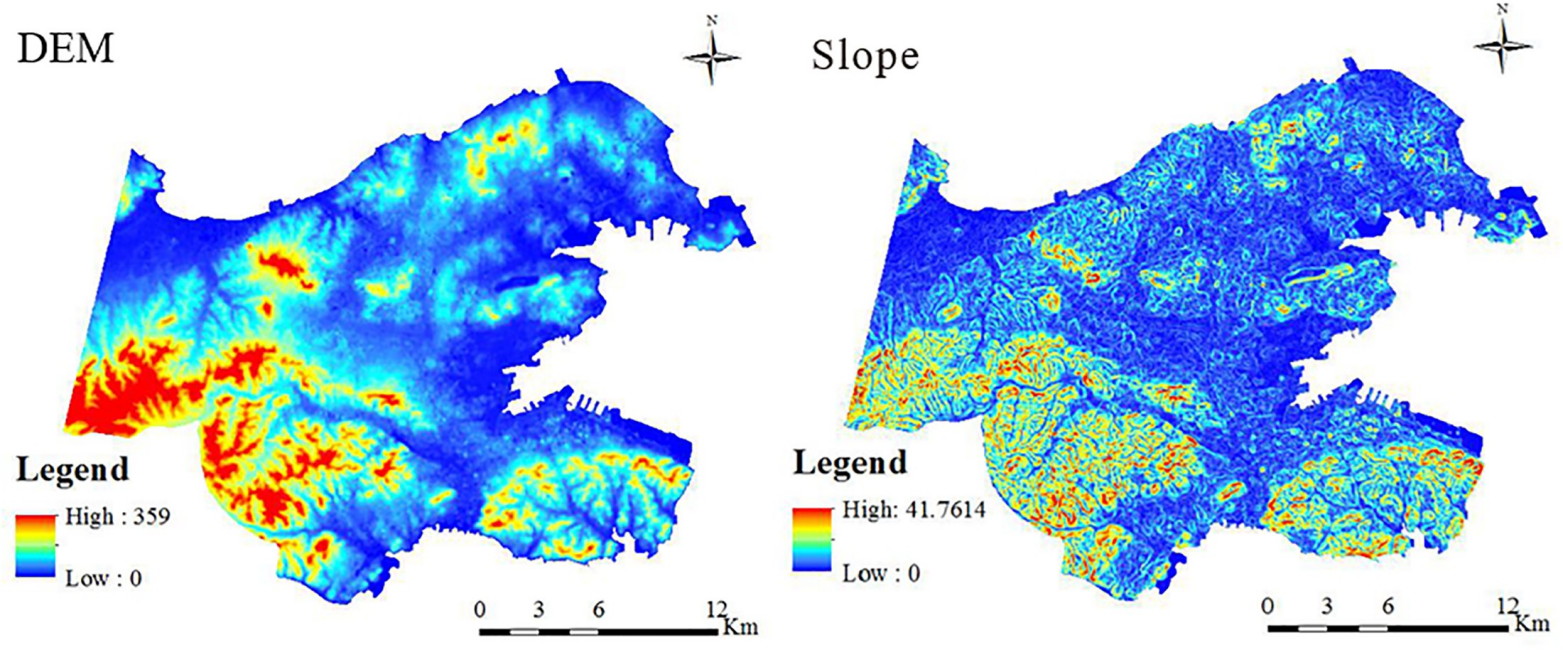
Elevation (DEM) and the slope of the study area.

**Fig 7:**
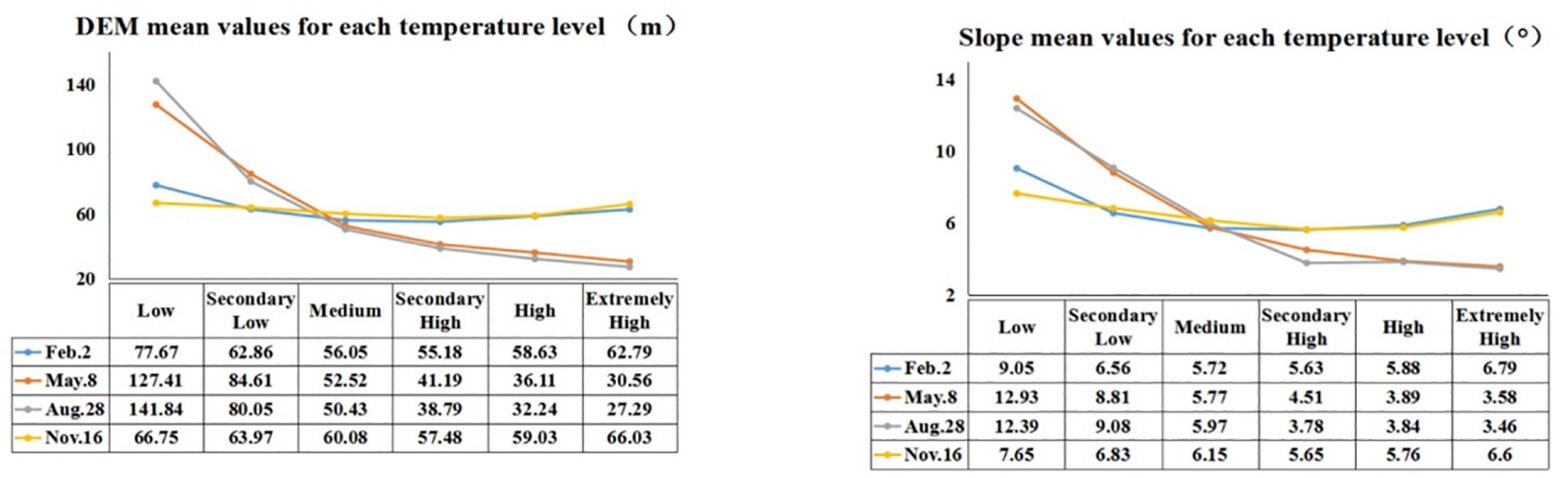
Mean elevation (DEM) and the slope values for each temperature level.

**Fig 8:**
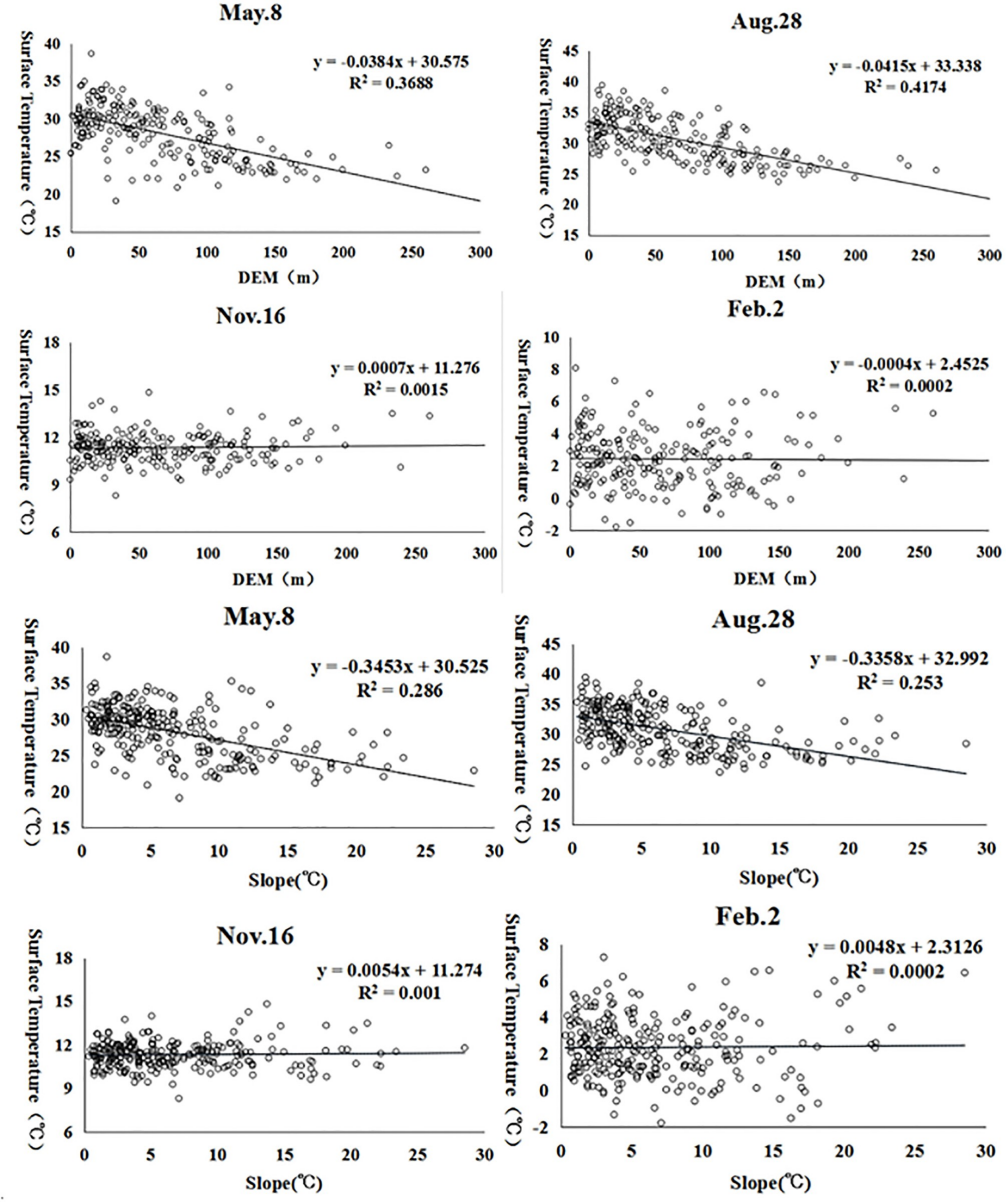
Scatter plot of the DEM, slope and surface temperatures.

The elevation and slope maps of Dalian (Fig 6) show that the southwestern region is occupied by low mountains with relatively higher elevations and slopes. Comparatively, other areas are occupied by plains with lower elevations and slopes. The relationships between the land surface temperature and the DEM and the slope are shown in Figs 7 and 8. Elevation and slope are significantly and negatively associated with land surface temperature in spring and summer. For autumn and winter, however, there is almost no correlation between the surface temperature and both the elevation and the slope.

#### NDVI

**Fig 9:**
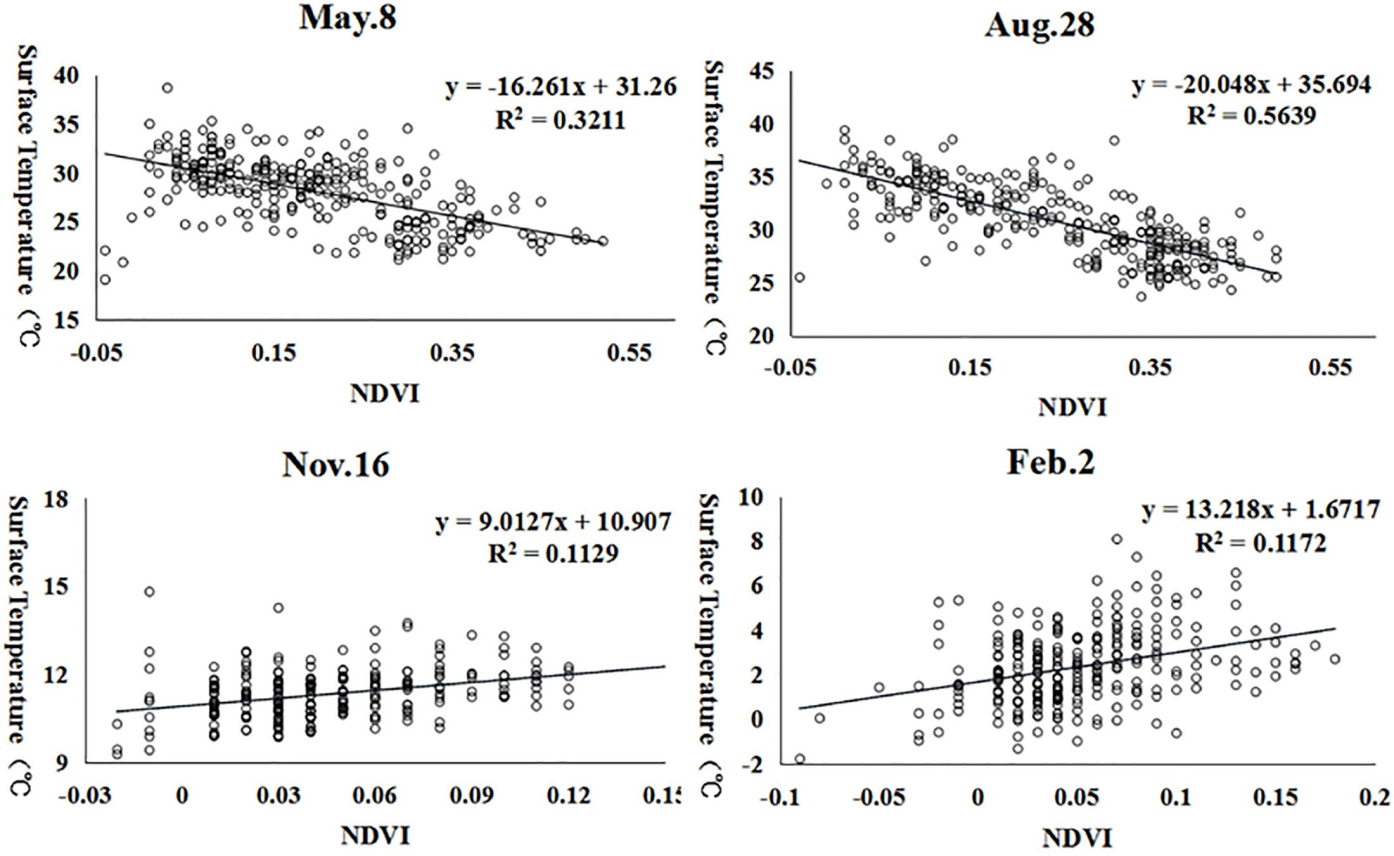
Scatter plot of the NDVI and the surface temperature.

In addition to the elevation and the slope, we also analyzed the effect of the vegetation cover on the UHI of Dalian City. Vegetation is considered to have a cooling effect and reduces the temperature of underlying surfaces. Most scholars have employed the NDVI to represent vegetation coverage. This method was also used in this study to analyze the relationship between the surface temperature and the vegetation coverage. The correlation analysis results show that the surface temperature negatively correlates with the NDVI in spring and summer and positively correlates with the NDVI in winter and autumn (Fig 9). The results indicate that the surface temperature decreases with increasing vegetation coverage in spring and summer and increases in autumn and winter.

#### Distance to the coastline

**Fig 10:**
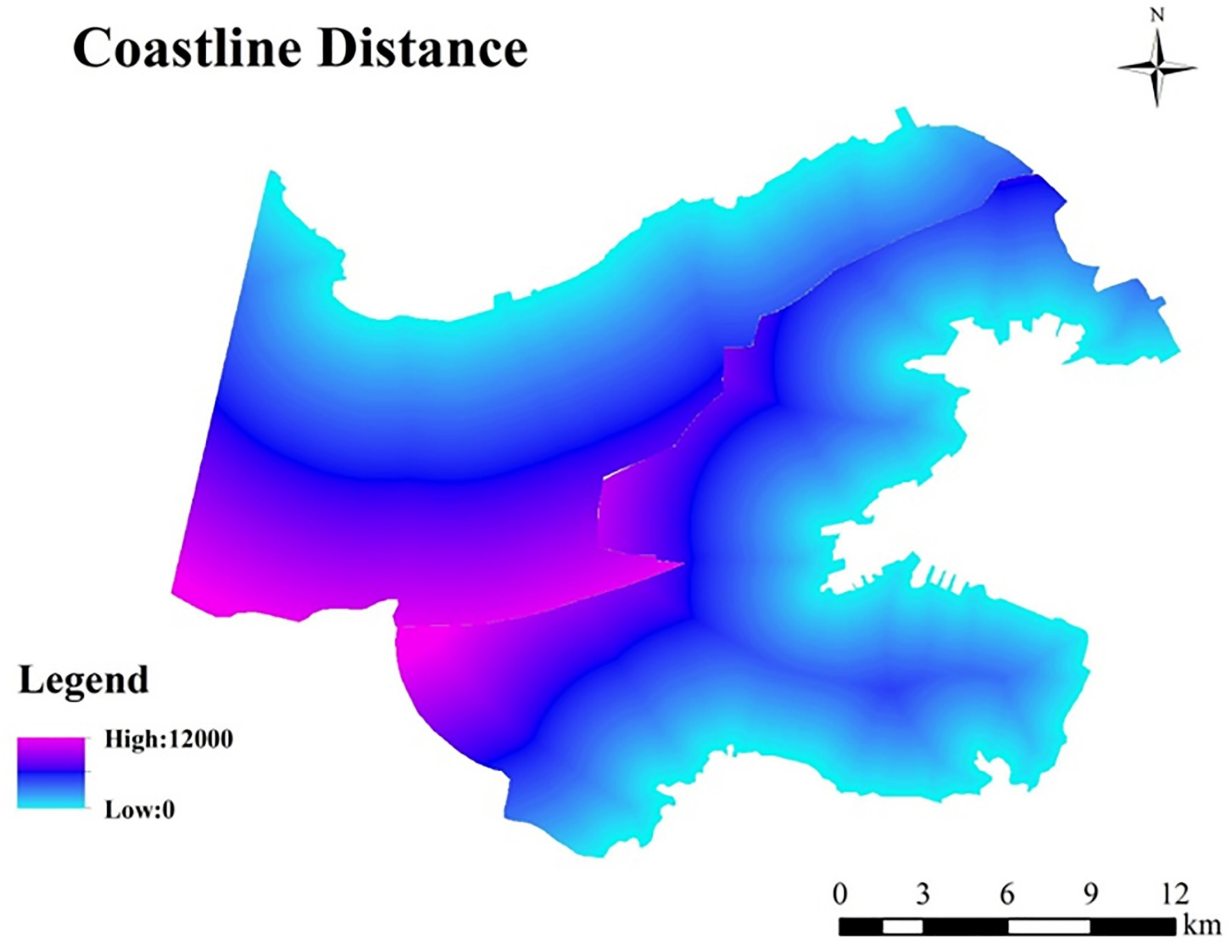
Distance to the coastlines.

From the coast to the inland, we created eight 1000 m buffers starting from the north shore and the east shore of the study area (Fig 10). The results suggest that the distance to the sea does not play a major role in the UHI effect, possibly due to the relatively small study area.

#### Stable lights

**Fig 11:**
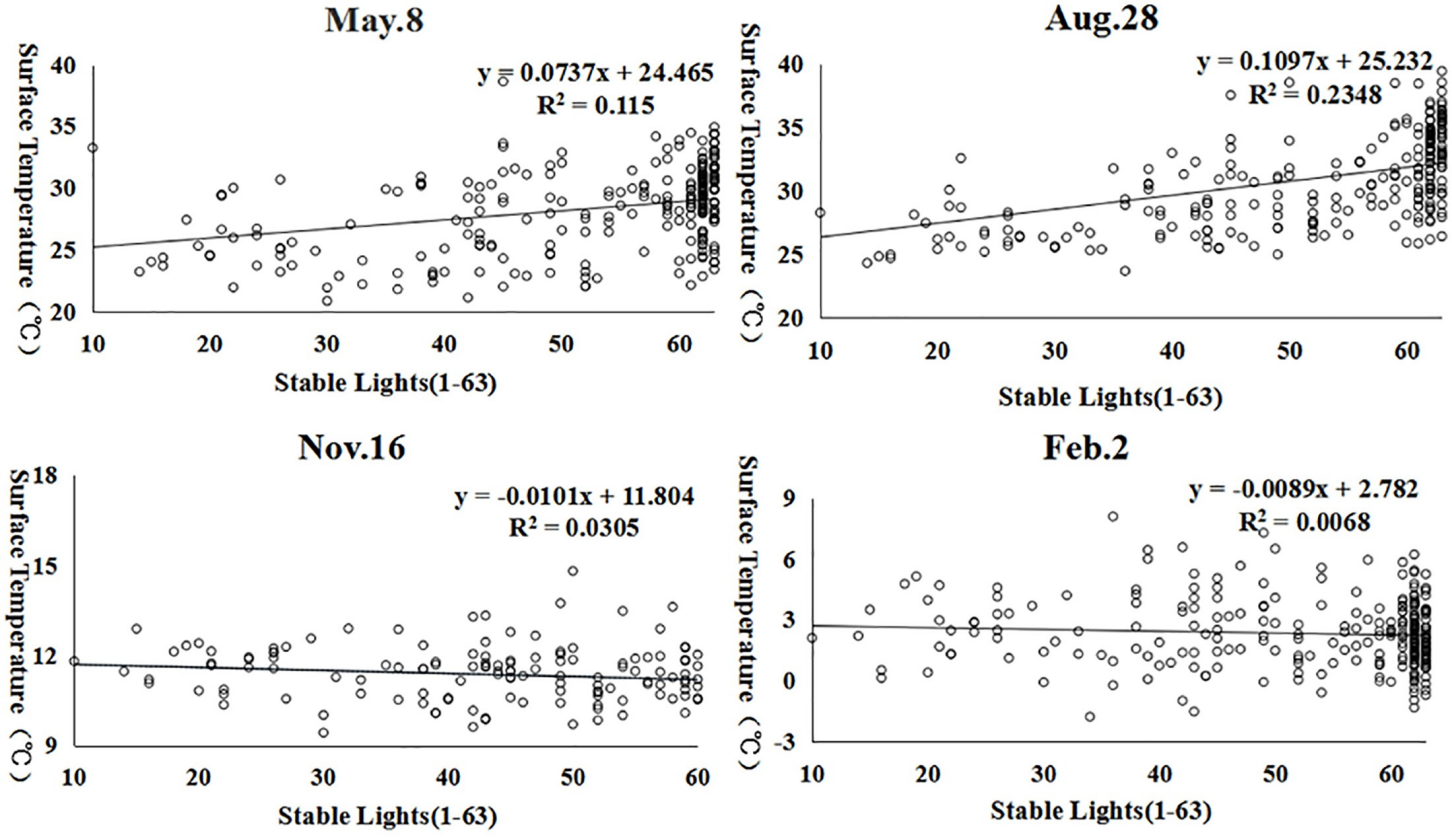
Scatter plot of the stable lights data and the surface temperature.

In addition, we employed stable lights data to represent the population distribution [27, 28] and analyzed the relationship between the surface temperature and the population distribution. The correlation analysis results showed that the surface temperature is positively correlated with the population distribution in spring and summer (Fig 11). That is, the surface temperature increases with increasing population, and this phenomenon is more obvious in summer. With the increase in population, more heat is generated because of the use of air conditioning systems, and there is no significant correlation between the winter surface temperature and the population distribution, which indicates that the effect of the population distribution on the winter surface temperature is small.

### Cubist results

In addition to simple correlation analysis, we further applied the cubist algorithm to analyze the effects of different factors on the surface temperature in different seasons. We randomly selected 200 points to examine the relationship between the surface temperature and other influential factors, including the elevation (DEM), slope, vegetation coverage (NDVI), and stable lights. The results (Table 5) suggest that these factors can adequately explain the UHI effect in spring and summer (with an R^2^ of 0.77 and 0.85, respectively) but perform poorly in autumn and winter. For individual factors, the vegetation coverage (NDVI) is clearly negatively associated with the land surface temperature in spring and summer and positively associated with the land surface temperature in autumn and winter. The elevation is consistently and negatively associated with the land surface temperature, and the relationship is much stronger in spring and summer. Moreover, the population distribution (e.g., stable lights) is only relevant in the summer, but the slope plays a role in spring and winter.

**Table 5:** Cubist analysis results.

Seasons	Equations	R^2^
Spring (May 8)	LST = 32.151–9.5NDVI—0.176Slope—0.0138DEM	0.77
Summer (Aug 28)	LST = 31.908–12.4NDVI-0.0209DEM+0.062Stable Lights	0.85
Autumn (Nov 16)	LST = 10.907+13.8NDVI-0.038DEM	0.41
Winter (Feb 2)	if NDVI>0.09, then LST = 1.742+8.4NDVI-0.027Slope-0.0019DEM If NDVI≦0.09, then LST = 1.469+37.2NDVI-0.103Slope-0.0004DEM	0.49

## Discussion

The UHI effect is essential in public health. Existing research on UHIs largely emphasizes inland cities, and little research has been performed to examine UHIs in coastal cities. Coastal cities, however, may illustrate significantly different UHI effects due to the impact of the sea. This research indicates that the sea does play a significant role in affecting the UHI effects in different seasons. That is, moderate UHI effects exist in spring and summer, while no UHI or urban cooling effects were found in autumn and winter. The distance to the coastline, however, does not have a significant effect on the land surface temperature. These results indicate that although the coastal city has been impacted by the sea, the spatial difference of such impact is insignificant at the local level and has little spatial impact on the urban heat island effect.

This research proves that seasonality plays a major role in the UHI effect. Past research always emphasizes the same season, without considering the UHI during different seasons. Vegetation coverage (NDVI) is clearly negatively associated with the land surface temperature in spring and summer and is positively associated with the land surface temperature in autumn and winter. The elevation is consistently negatively associated with the land surface temperature, and the relationship is much stronger in spring and summer. Moreover, the population distribution (e.g., stable lights) is only relevant in the summer season, and the slope plays a role in spring and winter. Such analyses with a nonparametric cubist regression tree model are important because seasonal variations play a major role in the urban heat island effect, because different factors affect the surface temperature during different seasons and at different magnitudes.

## Conclusions

In this paper, we examined the UHI effect and its seasonality in a coastal city (Dalian, China). Furthermore, influential factors, including the vegetation coverage, elevation, surface slope, distance to coastline, and population distribution, were identified, and their effects on land surface temperature were examined. The results suggest that the UHI effect only exists in spring and summer, and no obvious UHI effect can be found in autumn and winter. The adjacency to the sea leads to moderate UHI effects in spring and summer, and no UHI or urban cooling effect exists in autumn and winter. The distance to the coastline, however, does not play a role in the UHI effect. Furthermore, as one of the most important factors, the vegetation coverage plays a significant role in leading the UHI effect in spring and summer and has a significant role in mediating the UHI effect in autumn and winter. Comparatively, the elevation (e.g., digital elevation model (DEM)) is consistently negatively associated with the UHI in all seasons, although a stronger relationship was found in spring and summer. In addition, the surface slope is also a significant factor in spring and winter, and the population density also impacts the UHI distribution in summer. We also concluded that for the same area, seasonal variations played a major role in the urban heat island effect, as different factors affected the surface temperature during different seasons and at various magnitudes.

## Supporting information

S1 Data (ZIP)
